# Electrospun Polymer-Fungicide Nanocomposites for Grapevine Protection

**DOI:** 10.3390/polym13213673

**Published:** 2021-10-25

**Authors:** Nasko Nachev, Mariya Spasova, Petya Tsekova, Nevena Manolova, Iliya Rashkov, Mladen Naydenov

**Affiliations:** 1 Laboratory of Bioactive Polymers, Institute of Polymers, Bulgarian Academy of Sciences, Acad. G. Bonchev St., Bl. 103A, BG-1113 Sofia, Bulgaria; nachev_n@polymer.bas.bg (N.N.); cekovapetya@polymer.bas.bg (P.T.); manolova@polymer.bas.bg (N.M.); rashkov@polymer.bas.bg (I.R.); 2 Department of Microbiology, Agricultural University, BG-4000 Plovdiv, Bulgaria; mladen@au-plovdiv.bg

**Keywords:** biodegradable polymer, electrospinning, fungicide, *Phaeomoniella chlamydospora*, *Phaeoacremonium aleophilum*, esca

## Abstract

Nowadays, diseases in plants are a worldwide problem. Fungi represent the largest number of plant pathogens and are responsible for a range of serious plant diseases. Esca is a grapevine disease caused mainly by fungal pathogens *Phaeomoniella chlamydospora* (*P. chlamydospora*) and *Phaeoacremonium aleophilum* (*P. aleophilum*). The currently proposed methods to fight esca are not curative. In this study, polymer composites based on biodegradable polymer containing chemical fungicides with antifungal activity were successfully prepared by electrospinning. The obtained materials were hydrophobic with good mechanical properties. In vitro studies demonstrated that the fungicide release was higher from PLLA/K5N8Q fibrous mats (ca. 72% for 50 h) compared to the released drug amount from PLLA/5-Cl8Q materials (ca. 52% for 50 h), which is due to the better water-solubility of the salt. The antifungal activity of the fibrous materials against *P. chlamydospora* and *P. aleophilum* was studied as well. The incorporation of the fungicide in the biodegradable fibers resulted in the inhibition of fungal growth. The obtained materials are perspective candidates for the protection of vines from the penetration and growth of fungal pathogens.

## 1. Introduction

Grapevine trunk diseases reduce the lifespan of vineyards and increase the costs of producing wine grapes [1]. They are caused mainly by fungal pathogens, with the major pathogens including *Phaeomoniella chlamydosporum*, *Phaeoacremonium aleophilum*, *Botryosphaeria* spp., *Cylindrocarpon* spp., *Eutypa lata*, and *Phomopsis viticolaand* [2].

Esca is a destructive grapevine trunk disease first described over 100 years ago, which occurs worldwide and induces heavy economic losses [3]. The disease could be developed by intensive pruning, frost, and other mechanical injuries. The first symptoms of esca appear as dark red or yellow stripes on leaves, which eventually dry and become necrotic. The disease can then progress, potentially causing the entire plant to die [4]. Esca was first successfully controlled in 1903, when sodium arsenite was used as an insecticide on grapes. However, sodium arsenite was noted as being highly toxic and carcinogenic, and since 2003 has been banned in Europe. Nowadays, in practice, there is no curative approach for fighting esca. This fact challenges researchers to find a solution to effectively fight with this complex disease.

Electrospinning is a facile and efficient technique for fabricating functional nanofibrous materials possessing a large specific surface area and a fine porous structure [5,6]. These fibers have received much attention for use in many applications: Biomedical applications such as drug delivery [7], tissue engineering [8], and wound dressing, as well as cosmetics and functional materials and devices such as composite reinforcement, filters, protective clothing, and smart textiles, and even energy and electronics such as batteries/cells, capacitors, sensors, and catalysts.

Recently, novel varieties of the electrospinning technique were developed in order to generate more complex nanostructures with desired features such as coaxial electrospinning [9], side-by-side [10] and tri-axial [11] electrospinning, and other multiple fluid processes. Such methods of fabrication can lead to composite structures such as core–shell, Janus, tri-layer core–shell, and other complex structures. Another recently used strategy is to combine electrospinning with other traditional methods to fabricate novel nanofibers [12]. Regardless of the direction, the final objective is a suitable application of the resultant nanofibers [13]. The present study highlights a new potential application of electrospun nanofibers for grapevine protection.

Phytopathogenic fungal infections have become a serious problem in agricultural production, reducing food yield and quality. Therefore novel antifungal agents with high efficiency and low toxicity are needed. Modern plant protection products should be designed to achieve the desired biological effect without harmful impact or side effects. The use of nanomaterials in agriculture and, in particular, for the protection of vineyards is an emerging field of interest. Sett et al. created rayon membranes on which nanofibers of soy protein/polyvinyl alcohol and soy protein/polycaprolactone are electrospun. This material aims to physically block the penetration of fungal spores [14]. However, the authors commented that the blocking was insufficient and an antifungal component should be incorporated. Furthermore, electrospun materials from two copolymers loaded with polyhexamethylene guanidine have been fabricated to serve as bandages for vineyards against the penetration of esca-causing fungi [15]. The authors reported, however, that more effective polymers and antifungal agents should be used.

Some authors have also proposed an original approach and have successfully obtained fibrous materials from poly(3-hydroxybutyrate), nanosized TiO_2_-anatase, and chitosan oligomers (COS) with antifungal activity for plant protection via a combination of electrospinning and electrospraying [16]. The obtained eco-friendly materials possess high roughness, hydrophobicity, and antifungal activity against *P. chlamydospora*.

It is known that compounds containing 8-hydroxyquinoline exhibit anticancer [17,18], antimicrobial [19,20], antiviral [21], and antifungal activities [22,23]. Due to their antifungal properties, these biologically active compounds have found application in agriculture. We created stable solutions on the basis of a water-soluble polymer and a fungicide with antifungal activity suitable for applications in agriculture [24].

The incorporation of low molecular weight derivatives of 8-hydroxyquinoline into fibrous polymer materials obtained by electrospinning is of interest because it allows combining the valuable biological properties of 8-hydroxyquinoline derivatives with the advantages of electrospun materials. In our previous study, we obtained fibrous membranes on the basis on cellulose acetate loaded with an antifungal agent for active protection against spore penetration and plant infection in vineyards [25]. However, the incorporation of low molecular weight derivatives significantly lower the physicochemical properties and therefore they need to be improved.

In the last few decades, considerable research interest has been dedicated to the use of biodegradable polymer materials for various applications, such as in medicine, as well as in industry to replace conventional petrochemical-based polymers. Due to its merits, thermoplastic aliphatic polyesters are the most commonly explored synthetic biodegradable polymers. PLA has an extensive mechanical property profile and is a highly biocompatible and biodegradable polymer [26].

Therefore, the present study aimed to prepare electrospun composite materials from PLA and 8-hydroxyquinoline derivatives with antifungal activities. The effect of the incorporated biologically active compound on the morphology, wetting, crystallinity, thermal, and physicochemical properties was studied. Microbiological tests against *P. chlamydospora* and *P. aleophilum* were performed.

## 2. Materials and Methods

### 2.1. Materials

Poly(L-lactide) (PLA; Ingeo™ Biopolymer 4032D, NatureWorks LLC—USA, Minnetonka, MN, USA; M_W_ = 259,000 g/mol; M_W_/M_n_ = 1.94; as determined by size-exclusion chromatography using polystyrene standards), 5-nitro-8-hydroxyquinoline (Pharmachim, Sofia, Bulgaria), and 5-chloro-8-hydroxyquinoline (Sigma-Aldrich, St. Louis, MO, USA) were used. Dichloromethane (DCM; Merck, Darmstadt, Germany) and ethanol (abs. EtOH; Merck, Darmstadt, Germany) were of an analytical grade of purity.

Potato dextrose agar medium was obtained from Merck, Darmstadt, Germany. The disposable consumables were purchased from Orange Scientific, Braine-l’Alleud, Belgium.

### 2.2. Procedures

The potassium 5-nitro-8-hydroxyquinoline was prepared as described by Ermakov and coworkers [27].

### 2.3. Preparation of Electrospun Fibrous Materials

Spinning solutions in DCM/EtOH (DCM/EtOH = 90/10) were prepared for PLA, PLA/5-Cl8Q, and PLA/K5N8Q. The total polyester concentration was 10 wt% 5-Cl8Q and 10 wt% K5N8Q.

A Brookfield LVT viscometer equipped with an adaptor for small samples, a spindle, and a camera SC 4-18/13 R at 20 ± 0.1 °C was used to measure the solution viscosities. The spinning solutions were measured in triplicate and the mean values with their standard deviations were used.

Electrospinning was performed using a high-voltage power supply (up to 30 kV), a grounded metal drum collector, an infusion pump (NE-300 Just InfusionTM Syringe Pump, New Era Pump Systems Inc., Farmingdale, NY, USA) for delivering the spinning solution at a constant rate, and a syringe equipped with a metal needle (gauge: 20GX1½″). The applied voltage was 25 kV, the distance to the collector was 15 cm, the collector rotating speed was 1000 rpm, the humidity was 50%, and the temperature was 20 °C.

### 2.4. Characterization of the Electrospun Materials

The morphology of the fibers was examined by a scanning electron microscope (SEM). The samples were vacuum-coated with gold and observed by a Jeol JSM-5510 SEM (Jeol Ltd., Tokyo, Japan) at acceleration voltage of 10 kV with 1000×, 2500×, and 5000× magnification. The fiber morphology was evaluated using the criteria for complex evaluation of electrospun materials as described elsewhere [28] via ImageJ software by measuring the diameters of at least 20 fibers from each SEM micrograph [29].

Attenuated total reflection Fourier transform infrared (ATR-FTIR) spectra were recorded using an IRAffinity-1 spectrophotometer (Shimadzu Co., Kyoto, Japan) equipped with a MIRacle™ATR (diamond crystal with a depth of penetration of the IR beam into the sample of approximately 2 μm) accessory (PIKE Technologies, Fitchburg, WI, USA) in the range of 600–4000 cm^−1^ and a resolution of 4 cm^−1^. All spectra were corrected for H_2_O and CO_2_ using an IRsolution software program.

The absence/presence of a crystalline phase in the electrospun materials was assessed by X-ray diffraction analysis (XRD). XRD spectra were recorded at r.t. using a computer-controlled D8 Bruker Advance powder diffractometer (Bruker, Billerica, MA, USA) with a filtered CuKα radiation source and a luminescent detector. The analyses were performed in the 2θ range from 5° to 50° with a step of 0.02° and a counting time of 1 s/step.

Static contact angle measurements of the membranes were performed using an Easy Drop DSA20E Krűss GmbH drop shape analysis system (Hamburg, Germany) at 20 ± 0.2 °C. A sessile drop of deionized water with a volume of 10 μL controlled by a computer dosing system was deposited onto the electrospun fibrous materials. The contact angles were calculated by computer analysis of the acquired images of the droplet. The data are an average from 10 measurements for each sample.

Mechanical properties were evaluated by tensile measurements performed on the fibrous materials using a single-column system for mechanical testing INSTRON 3344, equipped with a loading cell 50 N and Bluehill universal software (Instron Bluehill Universal V4.05 (2017) software, Norwood, MA, USA). The stretching rate was 10 mm/min, the initial length between the clamps was 40 mm, and the room temperature was 21 °C. All samples were cut into dimensions of 20 × 60 mm^2^ with a thickness of ca. 200 µm. For the sake of statistical significance, 10 specimens of each sample were tested, after which the average values of Young’s modulus, the ultimate stress, and the maximum deformation at break were determined.

5-Cl8Q and K5N8Q release was studied in vitro at 37 °C in acetate buffer (CH_3_COONa/CH_3_COOH) containing lactic acid (acetate buffer/lactic acid = 96/4 *v*/*v*) at pH 3 and an ionic strength of 0.1. Fibrous materials loaded with 5-Cl8Q or K5N8Q (4 mg) were immersed in 100 mL of buffer solution under stirring in a water bath (Julabo, Seelbach, Germany). The release kinetics were determined by withdrawing aliquots (2 mL) from the solution at determined time intervals, then adding back the same amount of fresh buffer and recording the absorbance of the aliquots by a DU 800 UV–vis spectrophotometer (Beckman Coulter, Brea, USA) at wavelengths of 255 nm and 364 nm. The amount of released 5-Cl8Q or K5N8Q was calculated using calibration curves (correlation coefficient *R* = 0.999) for the membranes in acetate buffer/lactic acid = 96/4 *v*/*v* with a pH of 3 and an ionic strength of 0.1. The data are the average values from three measurements.

### 2.5. In Vitro Antifungal Assay

The antifungal activity of the fibrous materials was monitored against the fungi *P. chlamydospora* CBS 239.74 and *P. aleophilum* CBS 631.94. *P. chlamydospora* CBS 239.74 and *P. aleophilum* CBS 631.94 were purchased from Westerdijk Fungal Biodiversity Institute, Utrecht, the Netherlands.

*P. chlamydospora* and *P. aleophilum* grow normally on potato dextrose agar [30] and malt extract agar [31].

In the present study, in vitro studies were performed using potato dextrose agar medium (PDA; Merck, Germany). The surface of the solid agar was inoculated with a suspension of fungi culture with a fungi concentration of 1 × 10^5^ cells/mL, and on the surface of the agar in each Petri dish, one electrospun material (17 mm in diameter) was placed. The Petri dishes were incubated for 96 h for *P. chlamydospora* and *P. aleophilum* at 28 °C, and subsequently, the zones of inhibition around the disks were measured. The average diameters of the zones of inhibition were determined using ImageJ software based on 15 measurements in 15 different directions for each zone.

For the preparation of the conidia suspension test, microorganisms were grown on potato dextrose agar (PDA) medium for 14 days. Conidia were obtained by pouring 5 mL of sterile water onto the plate and washing it off with a sterile loop. Conidia suspensions were filtered through two layers of sterile round cloth to remove mycelial fragments. The final concentration of conidia was adjusted to 10^7^ conidia/mL with sterile water. The fibrous materials were cut into disks with a diameter of 45 mm and a thickness of ~1 µm. Digital Thickness Gauge FD 50 (Käfer GmbH, Villingen-Schwenningen, Germany) was used to determine the thickness of the fibrous materials. All fibrous materials were sterilized for 30 min under UV light in a laminar box before being used for further experiments. Then, the fibrous material in disk form was placed between the two parts of the filtration device supplied with a pump. The two parts of the device were pinched with a clip. After this, 20 mL of the spore conidia suspension was passed through each type of fibrous material. Then, every used disk was taken with pincers and placed on a surface of solid PDA medium in a Petri dish. The Petri dishes were maintained at 28 °C for 96 h. Then, the fungal growth was assessed. The concentration of conidia passed through the materials was determined using a hemocytometer.

### 2.6. Statistical Analysis

The data are displayed as means ± standard deviations (SDs). To determine the statistical significance of the data, one-way analysis of variance (ANOVA) followed by Bonferroni’s post-hoc test were performed. Values of * *p* < 0.05, ** *p* < 0.01, and *** *p* < 0.001 were considered significant.

## 3. Results and Discussion

### 3.1. Morphological Analysis

In our previous studies, we showed the successful fabrication of fibrous materials obtained from polyesters by electrospinning [20,32,33]. In concrete, we found that for PLA with M_W_ = 259,000 g/mol and M_W_/M_n_ = 1.94, the optimal total polymer concentration for conducting electrospinning resulting in preparation of defect-free cylindrical fibers was 10 wt% in a DCM/EtOH solvent system [20]. However, it is necessary to study the effect of incorporation of a biologically active substance(s) on the morphologies of fibers, their physical–chemical properties, and their ability to inhibit the growth and penetration of pathogenic fungi.

**Figure 1:**
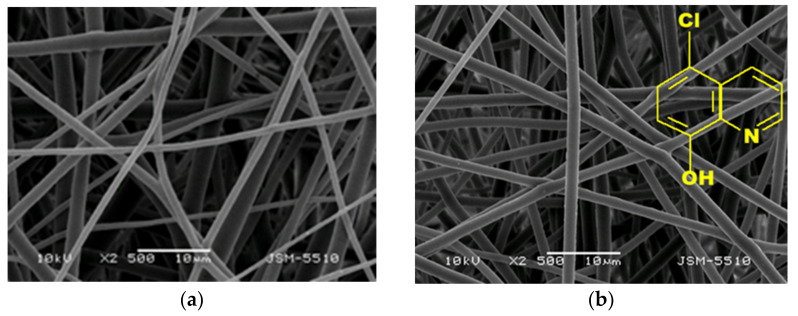

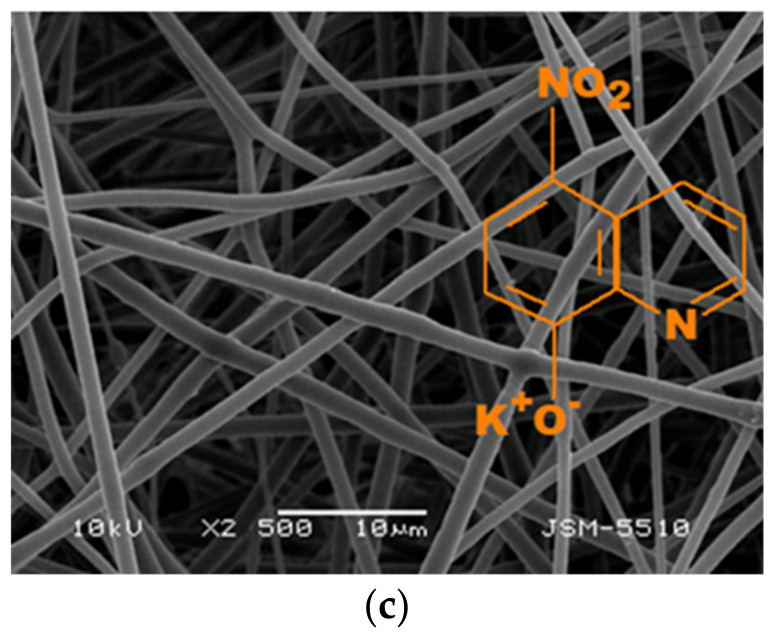
Representative SEM images of the fibers of electrospun fibrous materials of PLLA (**a**), PLLA/5-Cl8Q (**b**), and PLLA/K5N8Q (**c**); magnification ×2500.

SEM pictures of the obtained PLLA, PLLA/5-Cl8Q, and PLLA/K5N8Q fibers are presented in Figure 1. The electrospinning of PLLA resulted, reproducibly, in the fabrication of fibers with average fiber diameters of 1045 ± 320 nm (Figure 1a). The obtained diameters of the PLA fibers are in fairly good agreement with the literature data [34]. As can be easily seen using the selected conditions (concentration, solvent system, applied voltage, feeding rate, collector rotating speed, etc.), fibers with a cylindrical shape without defects and pores were obtained.

The addition of chemical fungicides (5-Cl8Q or K5N8Q) at a concentration of 10 wt% resulted in the preparation of stable solutions that did not alter the process of electrospinning and resulted in the fabrication of composite fibers with a cylindrical shape with a mean diameter close to that of the neat PLLA (Figure 1b,c). The average diameter of the fibers of the fibrous materials based on PLLA/5-Cl8Q and PLLA/K5N8Q was 1125 ± 300 nm and 1065 ± 250 nm, respectively. This is an indication that the addition of low molecular fungicides (derivatives of 8-hydroxyquinoline) did not lead to a significant change in the fiber morphology or diameters and distribution. These findings were confirmed by the measured values of the dynamic viscosities of the prepared solutions as well. The dynamic viscosity for PLLA, PLLA/5-Cl8Q, and PLLA/K5N8Q were relatively close and were 1350 cP, 1500 cP, and 1420 cP, respectively.

### 3.2. Contact Angle Measurements

**Figure 2:**
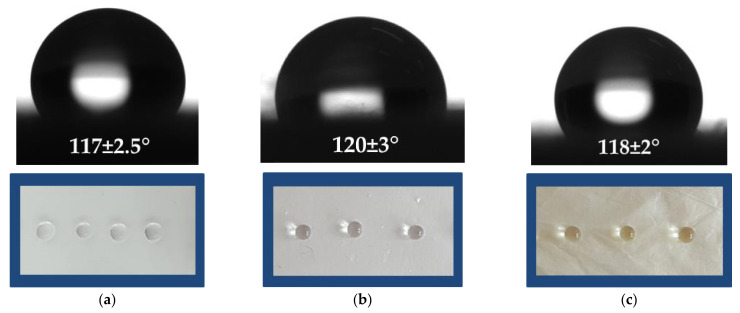
Images of water droplets deposited on the surface of fibrous materials: (**a**) PLLA, (**b**) PLLA/5-Cl8Q, and (**c**) PLLA/K5N8Q.

It is well known that bacterial and fungal adhesion is influenced by the surface characteristics and the hydrophilic/hydrophobic balance of the host surface. For this reason, it is important to determine the values of the contact angle of the prepared electrospun materials that will contact the fungal species. The values of the water contact angles for all obtained samples were determined using distilled water droplets, and representative images of the droplets are shown in Figure 2. The PLLA fibrous material was hydrophobic, with a water contact angle of 117° ± 2.5° (Figure 2a). The measured value for the pure PLLA was close to the values found in the literature [35]. The measured contact angle values of the PLLA/5-Cl8Q and PLLA/K5N8Q composite fibrous materials were 120° ± 3° and 118.0° ± 2°, respectively (Figure 2b,c). The measured water contact angle values were close to those measured for the PLLA electrospun material. All of the obtained and studied electrospun fibers had water contact angle values ca. 120° and were hydrophobic.

### 3.3. FTIR Spectroscopic Analysis

**Figure 3:**
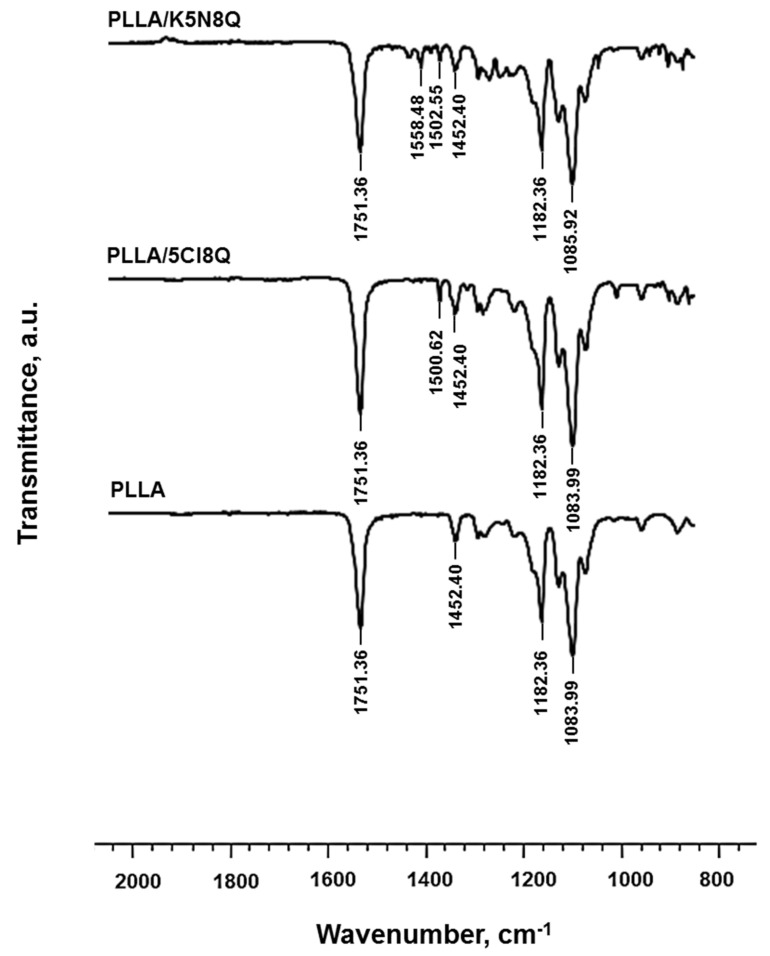
FTIR spectra of electrospun fibrous materials of PLLA, PLLA/5-Cl8Q, and PLLA/K5N8Q.

FTIR spectroscopy was performed to characterize the prepared PLLA, PLLA/5-Cl8Q, and PLLA/K5N8Q fibrous materials, and the recorded spectra are shown in Figure 3. Characteristic bands for PLLA appeared at 1751 cm^−1^ for the C=O groups and at 1182 cm^−1^ for the C–O–C groups.

The characteristic stretching frequencies for C–O at 1080 cm^−1^ and the bending frequencies for –CH_3_ asymmetric and –CH_3_ symmetric at 1452 cm^−1^ and 1361 cm^−1^, respectively, were identified, also in accordance with the literature [36].

A new band appeared at 1500 cm^−1^, characteristic for the aromatic ring of the chemical fungicide in the PLLA/5-Cl8Q and PLLA/K5N8Q fibrous materials (Figure 3), in addition to the characteristic bands of PLLA [37], proving its presence in the electrospun composite materials.

Clearly, no molecular interaction between PLLA and the used fungicides was detected on the FT-IR spectra of the PLLA/5-Cl8Q and PLLA/K5N8Q composite fibrous materials.

### 3.4. XRD Analysis

**Figure 4:**
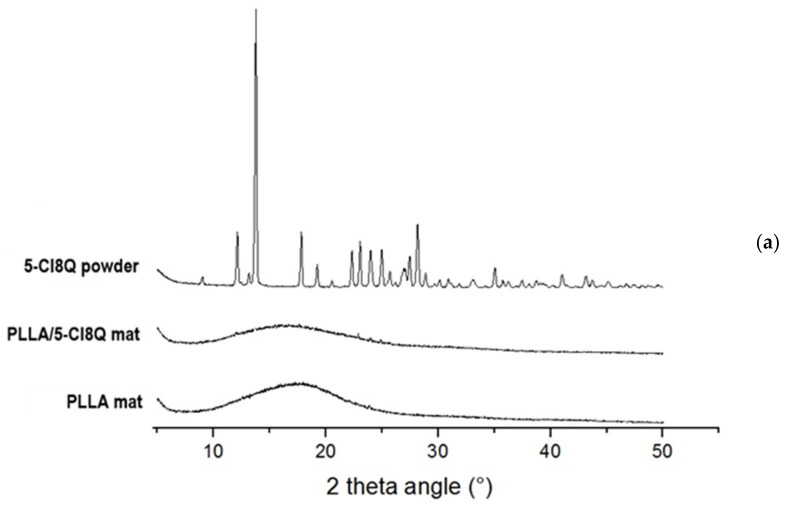

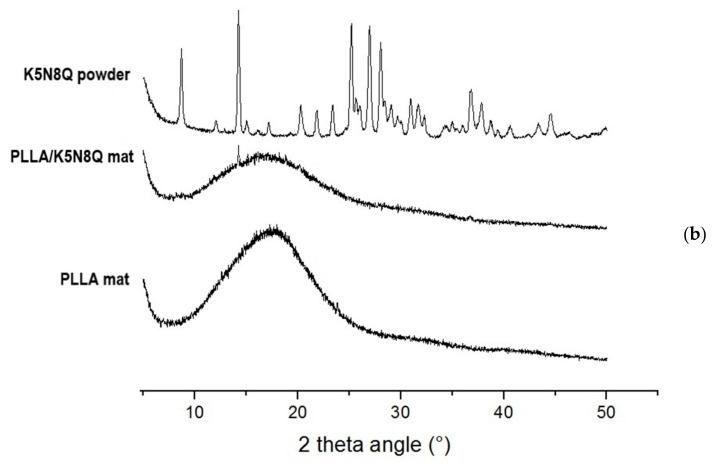
X-ray diffraction pattern of (**a**) 5-Cl8Q powder and PLLA and PLLA/5-Cl8Q materials, and (**b**) K5N8Q powder and PLLA and PLLA/K5N8Q materials.

Delivery systems based on nano- and microcarriers have been proven to be promising candidates for the delivery of poorly water-soluble or non-water-soluble compounds (drugs), wherein amorphization during thier encapsulation by the electrospinning process improves the dissolution of these compounds [12]. Therefore, it was of interest to study the changes in the crystallinity of chemical fungicides after their incorporation in composite electrospun fibrous materials. The crystallinity of the obtained fibers was determined by X-ray diffraction (XRD) analysis (Figure 4). The XRD pattern of PLLA and PLLA/5-Cl8Q materials and 5-Cl8Q powder, as well as of PLLA/K5N8Q fibrous material and K5N8Q powder, are presented in Figure 4a,b respectively. XRD patterns of 5-Cl8Q and K5N8Q (powder) with characteristic sharp diffraction peaks of the compounds were observed. These peaks showed that the fungicides (powders) were highly crystalline. The XRD spectra of the PLLA fibers showed a strong amorphous halo, proving that these materials have a typical amorphous structure. Moreover, in the spectra of the PLLA/5-Cl8Q and PLLA/K5N8Q composite materials, an amorphous halo was detected as well. This result indicates that each component in the composite fibrous materials prepared by electrospinning was in an amorphous state. This observation could be explained by the rapid drying of the jet during electrospinning, which impeded molecular motion. The obtained results are in accordance with the literature concerning the amorphization of poorly water-soluble drugs by electrospinning [38].

### 3.5. Mechanical Properties

**Figure 5:**
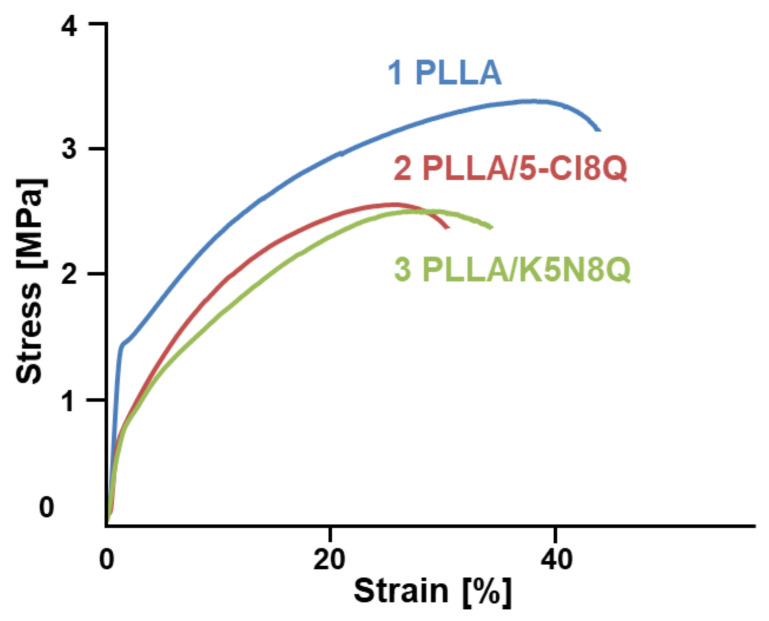
Stress–strain curves of electrospun materials: (1) PLLA, (2) PLLA/5-Cl8Q, and (3) PLLA/K5N8Q.

Stress–strain curves of the PLLA, PLLA/5-Cl8Q, and PLLA/K5N8Q electrospun materials are presented in Figure 5. The PLLA material showed the highest tensile strength values. The obtained values are in good agreement with the literature data [20]. The determination of the mechanical characteristics of the PLLA/5-Cl8Q and PLLA/K5N8Q composite fibers showed that these materials possess similar mechanical properties, albeit a bit lower than those of the PLLA fibrous materials. This result indicates that the incorporation of 5-Cl8Q in fibrous membranes does not considerably change the mechanical characteristics of these membranes. The tensile strength of the PLLA/5-Cl8Q and PLLA/K5N8Q composite fibrous materials was ca. 2.5 MPa, while the tensile strength of the PLLA fibrous materials reached 3.4 MPa. The slight decrease in mechanical characteristics might be due to the incorporation of low molecular weight chemical fungicides in the PLA matrix, which might have generated weak spots when the tensile test was carried out.

### 3.6. Cumulative Drug Release Analysis

The electrospinning method is often used for encapsulation of drugs for delivery. There are many data in the literature concerning the release rates of incorporated drugs in different polymer matrixes showing diverse behavior: Some show an initial burst release of the drug, while others show a more controlled release of the drug over a longer duration. This is due to the fact that many parameters could influence drug release, e.g., the molecular characteristics of the polymer, the polymer crystallinity and hydrophobicity, the nature of the drug and its crystallinity, the compatibility of the drug with the polymers matrix, the fiber morphology and diameters, the presence of defects along the fibers. Therefore, the same drug loaded in different polymer matrixes or different drugs incorporated in same polymer matrix may exhibit different release profiles.

**Figure 6:**
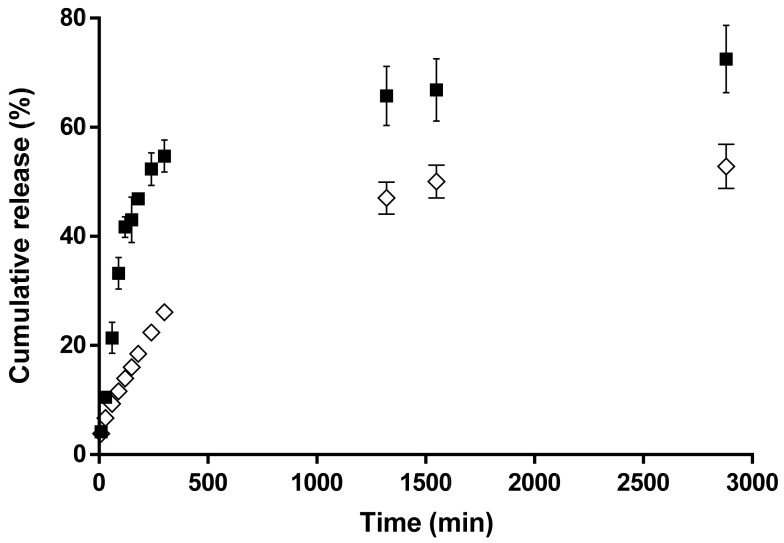
Release profiles of 5-Cl8Q and K5N8Q from PLLA fibers: PLLA/5-Cl8Q (◊) and PLLA/K5N8Q (■). The results are presented as the average values from three separate measurements with the respective standard deviation; acetate buffer/lactic acid (96/4 *v*/*v*), pH 3, 37 °C, ionic strength 0.1.

The release of 5-Cl8Q and K5N8Q from PLLA fibrous matrixes was studied, and their release profiles are shown in Figure 6. Initially, both drugs showed a relatively burst release from the PLLA fibrous matrix. However, K5N8Q was released in a higher amount compared to 5-Cl8Q for the same duration. For instance, the released K5N8Q was 10.5% and 21.4% for 30 and 60 min. For the same time durations, the released 5-Cl8Q was 6.7% and 9.3%, respectively. This difference in the release profiles could be due to the different natures of the drugs and their water solubility. K5N8Q is a partially water-soluble drug favoring a more rapid release. On the contrary, 5-Cl8Q is water-insoluble, which hampers its release. After 50 h, the amounts of the released 5-Cl8Q and K5N8Q were 52.8% and 72.5%, respectively.

### 3.7. Antifungal Activity of the Fibrous Membranes

The two main fungi causing esca disease are *P. chlamydospora* and *P. aleophilum*. The wounds on vines caused during pruning are the main point of entry for the penetration of fungal spores in grapevines. Because there is no direct way to fight esca, there is a rising demand for the development of novel plant protective agents and materials that are non-toxic but are efficient against esca.

Although there are some data with respect to the antifungal activity of 8-hydroxyquilonine derivatives against *Candida* species, there are no data concerning their effects on *P. chlamydospora* and *P. aleophilum*, which are the main causative agent of esca disease. In our previous study, we determined the minimum inhibitory concentration (MIC) of 5-Cl8Q against *P. chlamydospora* and *P. aleophilum*, and it was found to be 0.75 μg/mL for both strains [24]. The MIC determined by us for K5N8Q was 12.5 μg/mL and 25 μg/mL for *P. chlamydospora* and *P. aleophilum*, respectively.

The antifungal activity of the electrospun fibrous materials (diameter 17 mm) was determined by carrying out antifungal tests against *P. chlamydospora* and *P. aleophilum*.

**Figure 7:**
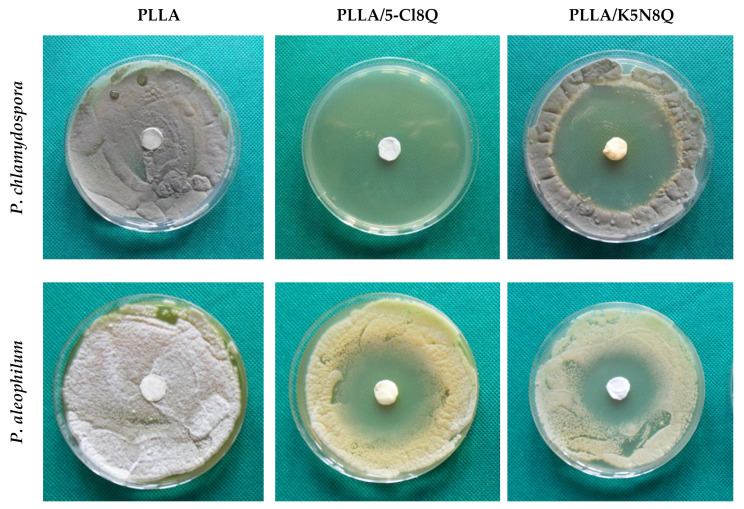
Digital pictures of the zones of inhibition against *P. chlamydospora* and *P. aleophilum* after contact of the fibrous materials with fungi cells. The material type is indicated at the top of each column. The cell type is marked in the left of each row.

Figure 7 presents the observed zones of inhibition after contact of the fibrous materials with the fungal cells. The loading of 5-Cl8Q in the composite fibrous materials that were laid in contact with *P. chlamydospora* resulted in complete inhibition of fungal growth. Moreover, there was a wide inhibition zone around the PLLA/5-Cl8Q disc put in contact with *P. aleophilum* (4.7 cm). Additionally, the incorporation of K5N8Q resulted in wide zones of inhibition as well. The diameters of the inhibition zones around the PLLA/K5N8Q discs were 6.2 cm and 4.0 cm against *P. chlamydospora* and *P. aleophilum*, respectively. From the obtained results, it is easily seen that *P. chlamydospora* is more vulnerable to treatment with the used 8-hydroxyquinoline derivatives.

The results obtained in the present study demonstrate that composite fibrous materials containing hydroxyquinoline derivatives have strong antifungal activity. In contrast, neat PLLA fibrous materials do not change the fungal growth or exhibit any antifungal activity.

**Figure 8:**
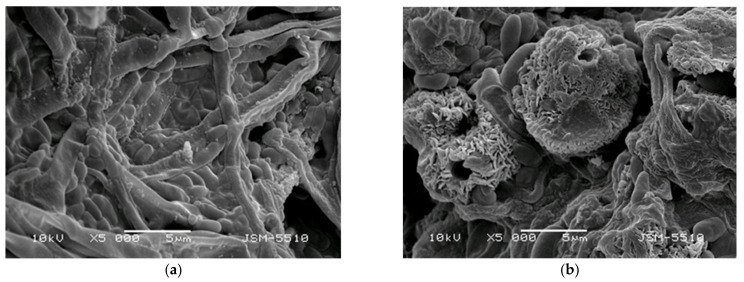
SEM micrographs of the (**a**) *P. chlamydospora* and (**b**) *P. aleophilum* conidia.

In the present study, the barrier efficacy of PLLA, PLLA/5-Cl8Q, and PLLA/K5N8Q electrospun fibrous materials were studied as well. For this purpose, 20 mL of conidia suspension was passed through each fibrous material (diameter 45 mm) using a filtration device. Initially, we determined the size of the *P. chlamydospora* and *P. aleophilum* conidia in the fungal suspension using SEM analysis. Figure 8 presents the used conidia.

The diameter and length of the *P. chlamydospora* conidia were ~0.75–1.2 µm and 1.8–2.3 µm, respectively. The measured diameter and length of the *P. aleophilum* conidia were ~1.1–1.5 µm and 2.5–3.5 µm, respectively. The initial concentration in the filtration experiments was 1 × 10^7^ conidia/mL for both strains. After passing through the electrospun discs, the determined spore concentration was 1.6 × 10^3^, 1.3 × 10^3^, and 1.4 × 10^3^ for the PLLA, PLLA/5-Cl8Q, and PLLA/K5N8Q materials, respectively. This result reveals that the final conidia concentration decreased significantly. However, some conidia passed through all of the fibrous materials in this study.

**Figure 9:**
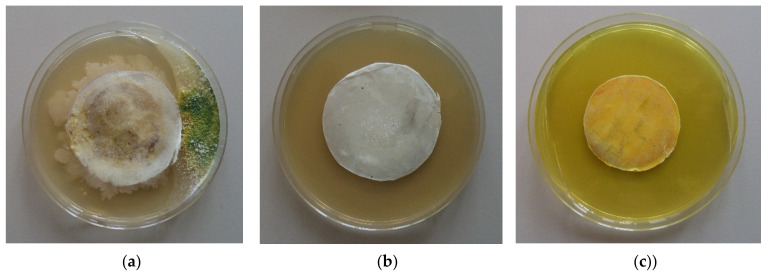
Digital images of the growth *P. chlamydospora* on the fibrous materials after spore filtration: (**a**) PLLA, (**b**) PLLA/5-Cl8Q, and (**c**) PLLA/K5N8Q.

It was of interest to determine not only the ability of the materials to impede the penetration of fungal spores, but also to study if the fibrous materials impede the growth of pathogenic fungi remaining in the material after filtration. Therefore, after filtration, we placed the used disks on a surface of solid agar in a Petri dish in order to determine the growth of the remaining fungi in the fibrous discs. The Petri dishes were incubated for 96 h at 28 °C, and then the fungal growth was determined. Figure 9 presents the growth of *P. chlamydospora* on the fibrous materials’ surface. It was found that the PLLA fibrous material used in the filtration experiments developed colonies of *P. chlamydospora* (Figure 9a). The developed colonies showed that this material did not possess antifungal activity. PLLA/5-Cl8Q and PLLA/K5N8Q, which were placed in suitable conditions for the development of remaining spores in the materials, impeded the fungal growth, resulting in compete fungal inhibition (Figure 9b,c).This result indicates that the fungi remaining in the PLLA/5-Cl8Q and PLLA/K5N8Q materials after filtration could not grow due to the antifungal activity of the obtained composite materials.

## 4. Conclusions

Novel micro- and nanofibrous materials of PLLA, PLLA/5-Cl8Q, and PLLA/K5N8Q were successfully electrospun. The obtained composite materials were hydrophobic with good mechanical properties. The incorporation of 5-Cl8Q or K5N8Q into the PLLA fibers imparted to them a considerable antifungal activity against *P. chlamydospora* and *P. aleophilum*. These features demonstrate that the obtained composite fibrous materials could be a potential candidate for application in agriculture for grapevine protection against esca-associated fungi.

## Data Availability

The data presented in this study are available on request from the corresponding author.
